# Optimised machine learning for time-to-event prediction in healthcare applied to timing of gastrostomy in ALS: a multi-centre, retrospective model development and validation study

**DOI:** 10.1016/j.ebiom.2025.105962

**Published:** 2025-10-10

**Authors:** Marcel Weinreich, Harry McDonough, Mark Heverin, Éanna Mac Domhnaill, Nancy Yacovzada, Iddo Magen, Yahel Cohen, Calum Harvey, Ahmed Elazzab, Sarah Gornall, Sarah Boddy, James J.P. Alix, Julian M. Kurz, Kevin P. Kenna, Sai Zhang, Alfredo Iacoangeli, Ahmad Al-Khleifat, Michael P. Snyder, Esther Hobson, Adriano Chio, Andrea Malaspina, Andreas Hermann, Caroline Ingre, Juan Vazquez Costa, Leonard van den Berg, Monica Povedano Panadés, Philip van Damme, Phillipe Corcia, Mamede de Carvalho, Ammar Al-Chalabi, Eran Hornstein, Eran Elhaik, Pamela J. Shaw, Orla Hardiman, Christopher McDermott, Johnathan Cooper-Knock

**Affiliations:** a Sheffield Institute for Translational Neuroscience (SITraN), University of Sheffield, UK; b Department of Clinical Neurobiology at the German Cancer Research Center (DKFZ) and the Medical Faculty of the Heidelberg University, Heidelberg, Germany; c Academic Unit of Neurology, Trinity College Dublin, Ireland; d Department of Molecular Genetics and Molecular Neuroscience, Weizmann Institute of Science, Rehovot, Israel; e Department of Neurology, Brain Center Rudolf Magnus, University Medical Center Utrecht, Utrecht, the Netherlands; f Department of Epidemiology, University of Florida, Gainesville, FL, USA; g Department of Basic and Clinical Neuroscience, Institute of Psychiatry, Psychology and Neuroscience, King's College London, London, UK; h Center for Genomics and Personalized Medicine, Stanford University School of Medicine, Stanford, CA, USA; i Rita Levi Montalcinì Department of Neuroscience, University of Turin, Turin, Italy; j UCL Queen Square MND Centre, Institute of Neurology; k Translational Neurodegeneration Section “Albrecht Kossel”, Department of Neurology, University Medical Center Rostock, University of Rostock, 18147, Rostock, Germany; l German Center for Neurodegenerative Diseases (DZNE) Rostock/Greifswald, 18147, Rostock, Germany; m Department of Neurology, Karolinska University Hospital, Stockholm, Sweden; n Department of Clinical Neurosciences, Karolinska Institutet, Stockholm, Sweden; o MND and Neuromuscular Research Unit, Department of Neurology, Hospital Universitario y Politécnico La Fe and Instituto de Investigación Sanitaria la Fe (IIS La Fe), Valencia, Spain; p Department of Medicine, University of Valencia, Valencia, Spain; q Centro de Investigación Biomédica en Red de Enfermedades Raras (CIBERER), Spain; r Hospital Universitari de Bellvitge–IDIBELL, L'Hospitalet de Llobregat, Barcelona, Spain; s Neurology Department, University Hospitals Leuven, Leuven, Belgium; t Neuroscience Department, Leuven Brain Institute, University of Leuven (KU Leuven), Leuven, Belgium; u Centre de Référence Maladies Rares SLA, CHU Tours, Tours, France; v UMR 1253 iBrain, Université de Tours, Inserm, Tours, France; w Instituto de Fisiologia, Centro de Estudos Egas Moniz, MUHCRI, Faculdade de Medicina, Universidade de Lisboa, Lisbon, Portugal; x Department of Neurosciences, King's College Hospital, London, United Kingdom; y Department of Biology, Lund University, Sweden; z Department of Neurology, Beaumont Hospital, Dublin, Ireland; aa FutureNeuro Research Ireland Centre for Translational Brain Science, Trinity College Dublin; ab NIHR Sheffield Biomedical Research Centre, Royal Hallamshire Hospital, Glossop Road, Sheffield, S10 2JF, UK

**Keywords:** Time-to-event prediction, Machine learning, Personalised medicine, Amyotrophic lateral sclerosis (ALS), Gastrostomy

## Abstract

**Background:**

Amyotrophic lateral sclerosis (ALS) is invariably fatal but there are large variations in the rate of progression. The lack of predictability can make it difficult to plan clinical interventions. This includes the requirement for gastrostomy where early or late placement can adversely impact quality of life and survival.

**Methods:**

We designed a model to predict the timing of gastrostomy requirement in ALS as indicated by 5% weight loss from diagnosis. We considered >5000 different prediction model configurations including spline models and a set of deep learning (DL) models designed for time-to-event prediction. The optimal prediction model was chosen via a Bayesian framework to avoid overfitting. Model covariates were measurements routinely collected at diagnosis; a separate longitudinal model also incorporated weight at six months. We employed a training dataset of 3000 patients from Europe, and two external validation cohorts spanning distinct populations and clinical contexts (United States, n = 299; and Sweden, n = 215). Missing data was imputed using a random forest model.

**Findings:**

The optimal model configuration was a logistic hazard DL model. The optimal model achieved a median absolute error (MAE) between predicted and measured time of 3.7 months, with AUROC 0.75 for gastrostomy requirement at 12 months. To increase accuracy we updated predictions for those who had not received gastrostomy at six months after diagnosis: here MAE was 2.6 months (AUROC 0.86). Combining both models achieved MAE of 1.2 months for the modal group of patients. Prediction performance is stable across both validation cohorts. Missing data was imputed without degrading model performance.

**Interpretation:**

To enter routine clinical practice a prospective study will be required, but we have demonstrated stable performance across multiple populations and clinical contexts suggesting that our prediction model can be used to guide individualised gastrostomy decision making for patients with ALS.

**Funding:**

Research Ireland (RI) and Biogen have supported the PRECISION ALS programme.

**Research in context**

**Evidence before this study**

Amyotrophic lateral sclerosis is an incurable and rapidly progressive neurodegenerative disease. Significant morbidity results from failure of adequate nutrition, typically due to weakness of muscles required for feeding and swallowing. Current practice is to overcome this barrier via placement of gastrostomy for parenteral feeding. Importantly the timing of gastrostomy has an impact on prognosis where late intervention after excessive weight loss is associated with shorter survival. We searched MEDLINE for studies published in English between Jan 1 2020 and Jan 1 2025 which included “amyotrophic lateral sclerosis”, “gastrostomy” and “prediction” in the title or abstract. Each of the studies identified applied one or two pre-specified models rather than seeking to survey a large number of models for the optimal approach. Each of the studies involved training in a single population-specific cohort and the majority did not feature external validation. Imputation of missing data was not performed in the majority of studies. No study included individualised quantitative predictions for the precise timing of gastrostomy.

**Added value of this study**

We provide a new model for prediction of time from diagnosis to requirement of gastrostomy for patients with ALS, with best-in-class performance across a range of metrics including sensitivity, specificity and the absolute error in prediction accuracy. We provide optimised model selection, hyperparameter tuning and imputation of missing data. Importantly we have optimised imputation of missing data and hyperparameter tuning to maximise utility and to avoid overfitting, even for deep learning models. Model performance is demonstrated by training and testing in multiple cohorts including two different external validation cohorts sourced from distinct populations and clinical contexts.

Additionally we present ‘predicTTE’, which is a customisable ‘app’ and accompanying online portal for any time-to-event analysis in any disease. Our tool enables a researcher with a dataset but no bioinformatics experience to design an optimal prediction model and make it available via an online portal to end-users within a ‘data-secure’ environment. We also provide the capacity for end-users to directly contribute additional training data.

**Implications of all the available evidence**

Our tool can be used to predict the timing of future gastrostomy for patients with ALS at the point of diagnosis. To enter clinical practice a prospective study will be required, but we have demonstrated stable model performance across multiple populations and clinical contexts. Our tool and online platform could be used to implement a similar strategy in other diseases.

## Introduction

Amyotrophic lateral sclerosis (ALS) is an incurable neurodegenerative disease where death results from motor neuron (MN) loss leading to respiratory failure. Relative to other neurodegenerative diseases ALS is rapidly progressive with the majority of patients surviving <5 years from diagnosis.^1^ Consequently the clinical course of ALS is largely a function of disease progression rather than other factors such as frailty and co-morbidities; this raises the possibility that, more than for other neurodegenerative diseases, ALS could be predictable based on measurements made at diagnosis.

Nutritional status and weight loss are independent prognostic factors for survival during the course of ALS.^2^ Dysphagia and limb weakness, combined with loss of appetite^3^ and respiratory effort,^4^ limit the utility of dietary modification to avoid weight loss. Indeed, current recommended clinical practice is that gastrostomy should be considered for all patients with ALS^5^ who reach >5% weight loss from diagnosis.^6^ Gastrostomy can stabilise weight loss in ∼50% of patients.^6^ Late gastrostomy often does not stabilise weight loss, and is also associated with higher periprocedural mortality.^2^ The decision to have a gastrostomy is complex and multifactorial, but uncertainty about timing means that the decision is often made late.^7^^,^^8^ Timing of gastrostomy is also relevant for clinical trials: Broadly predictions regarding the rate of disease progression can be used to stratify patients and so improve statistical power by reducing heterogeneity between test and placebo groups.^9^ More specifically, investigational medicinal products are often given orally and therefore the need for gastrostomy can be an exclusion criteria. In summary there is a compelling case for the need for accurate prediction of the timing for gastrostomy requirement.

**Fig. 1:**
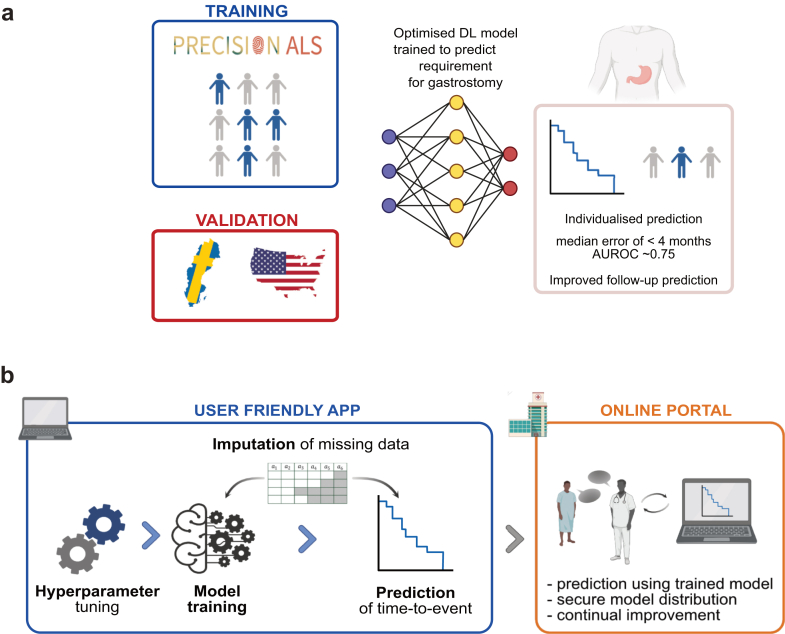
**Accessible and optimal time-to-event prediction of gastrostomy in ALS is achieved through implementation of state-of-the-art machine learning models within an app and online platform**. (**a**) We have developed an optimal deep learning (DL) model for prediction of time from diagnosis to requirement of gastrostomy which achieves a clinically actionable performance that is stable across multiple external validation cohorts from different populations and clinical contexts. (**b**) To develop our model we implemented a set of DL and spline models for time-to-event modelling. Our pipeline includes model selection, hyperparameter tuning, model training and imputation of missing data. This functionality is provided within a fully customisable ‘app’ (left panel). An accompanying online portal includes capacity for end-users including clinicians and researchers, to access and perform predictions using a trained model, and to contribute new data for model improvement, all within a data-secure environment (right panel).

We provide a clinical tool to predict optimal timing for gastrostomy in patients with ALS. We used an objective standard measure to indicate gastrostomy requirement: 5% weight loss from diagnosis.^6^ Cox regression^10^ is a popular model for time-to-event tasks which assumes a fixed proportional-hazard ratio, whereby the relative hazard-rate between patients is invariable over time. This is an unrealistic assumption for many contexts and has likely led to misinterpretation of the underlying drivers of time-to-event. Instead, to predict time until gastrostomy requirement, we tested >5000 different prediction model configurations including spline models^11^ and non-linear deep learning (DL) models designed for time-to-event prediction.^12^ We show that an optimal non-linear DL model is superior in our prediction task, particularly in the accuracy of individualised predictions as opposed to purely discriminative measures such as concordance. Model training used 3000 patient profiles from within the PRECISION^13^ dataset, sourced from eight sites across Europe. Model performance is stable in internal cross-validation and in two external independent cohorts from Sweden and the United States. Effective imputation means that model performance is robust to missing data. Our approach to prediction for timing of gastrostomy is summarised in Fig. 1a.

To ensure widespread usability we have implemented our model within an ‘app’ and accompanying online platform. The online platform includes the capacity to provide end-users such as clinicians, with secure access to the trained model for prediction, and with the option to contribute new data to improve model training. Moreover, our app is flexible and can be used to find an optimum model to make predictions for *any longitudinal event in any disease*; this functionally is automated end-to-end requiring no computational ‘expertise’. We have named our software ‘predicTTE’ (predicting time-to-event). Our tool is summarised in Fig. 1b.

## Methods

### Study cohorts

#### PRECISION

The patients with ALS included in this study as part of the PRECISION cohort were recruited at specialised neuromuscular centres in the UK, Belgium, Germany, Ireland, Italy, Spain, Turkey, and the Netherlands.^13^ We removed all patients who received gastrostomy within 30 days of diagnosis on the basis that they were likely to meet criteria for requirement of gastrostomy at baseline and therefore prediction was not necessary. We also removed patients where the first measured weight was more than three months distant from the date of diagnosis. This left n = 3000 patients which were used for model training. An additional PRECISION cohort recruited in Sweden became available through the course of this project and was added as an external validation cohort (n = 215).

#### PRO-ACE

The patients with ALS included in this study as part of the PRO-ACE external validation cohort were recruited at sites in the United States (US).^14^ Removal of patients who required gastrostomy at baseline left n = 299 patients.

Sex and race/ethnicity data were collected by researchers at each participating institution.

Longitudinal measurements of weight were used to calculate time to requirement for gastrostomy as indicated by 5% weight loss from diagnosis.^6^ To avoid confounding by the effect of gastrostomy, all weight measurements performed after the placement of gastrostomy as indicated by the ALSFRS-R score were removed. Moreover, to minimise confounding by inaccurate measurements of weight we determined the distribution of weight change over time and removed all measurements above the 95-percentile indicating an implausible rate of change in weight. In particular this included incidences where multiple different weight measurements were recorded at the same time point. A similar process was used to remove implausible outliers for all covariates.

### Software availability

The predicTTE online portal (https://www.predictte.org/) includes instructional material and links to download the app for different platforms.

### Ethics

The study was approved by the South Sheffield Research Ethics Committee (REC reference: 21/YH/0093; Project ID: SMNDD-020). Similarly this study followed study protocols approved by Medical Ethical Committees for each of the participating institutions: For PRECISION ALS this encompasses specialised neuromuscular centres in the UK, Belgium, Germany, Ireland, Italy, Spain, Turkey, and the Netherlands.^13^ PRO-ACE patients were recruited at sites in the United States (US).^14^ Written informed consent was obtained from all participating individuals. Details of procedures for handling of participant data within PRECISION ALS are found on the relevant web page (https://www.precisionals.ie/about/participant-privacy-information/). All PRO-ACE data is sourced from ERB/IRB approved observational studies, retrospective clinical assessments, and population registries. All methods were performed in accordance with relevant national and international research ethics guidelines and regulations, including the CIOMS 2016 guidelines. Data protection complied with the EU General Data Protection Regulation (GDPR) and, where applicable, the US Health Insurance Portability and Accountability Act (HIPAA).

### Statistics

#### Hyperparameter tuning and model choice

Model choice depends on the specific outcome measure; popular outcome measures used in prediction models include concordance, area under the cure of the receiver operating characteristic curve (AUROC) for a binary outcome, negative log-likelihood which is a measure of goodness-of-fit for model predictions based upon a probability distribution, and the related Brier score which applies a similar principle specifically to binary outcomes. However, while these measures may perform well in a *discrimination* between individuals with different risk of event, they do not necessarily achieve adequate *calibration*, which is a measure of how well the predicted risk of hazard matches the observed risk. Calibration is essential for a clinical prognosis where relative risk is not informative, although relative risk may be more important in the context of clinical trial stratification. Neither concordance or AUROC consider calibration. The Brier score theoretically considers both discrimination and calibration but fails to achieve good sensitivity at the cost of specificity if prevalence is low.^15^ We considered the median absolute error between observed and predicted time to event (MAE), but this has similar pitfalls to the Brier score; we have observed that models which optimise MAE tend to introduce a systematic bias which can compromise sensitivity. Our solution to this problem is to enable model choice and hyperparameter tuning with differential weighting of multiple outcome measures and in doing so we optimise both discrimination and calibration (Supplementary Figs. S2 and S3). Exhaustive hyperparameter tuning testing all possible model configurations is computationally intensive and liable to overfitting; to avoid this we implemented a Bayesian framework for model tuning^16^ (Supplementary Fig. S2).

Model choice and hyperparameter tuning were guided by comparative testing of model configurations encompassing the full range of parameters within the pycox^12^ implementation of MTLR,^17^ PC-Hazard,^18^ PMF,^18^ logistic hazard,^19^ DeepSurv,^20^ CoxTime,^12^ CoxCC,^12^ and DeepHit^21^ DL models. Model scheme and layer structure were prioritised based upon an initial grid search followed by a Bayesian optimisation of all other parameters^16^ (Supplementary Fig. S2). A Bayesian approach reduces the number of iterations and the potential for overfitting, particularly the use of an including an AdamWR optimiser with decoupled weight decay in the final stage (Supplementary Fig. S2). Hyperparameter tuning was performed via nested cross-validation: We separated the data into 80% for training and 20%, which we designate the testing dataset, for the final assessment of model performance. We then performed 10-fold cross validation within the 80% of data designated for training; here the data was *further* divided, on 10 separate occasions, into 80% for training and 20% for validation. The final prediction is output as the median of the 10 different predictions and compared with the testing dataset. We selected a random starting seed for each of the 10 rounds of cross-validation.

Similarly we tested 20 flexible parametric models including the Royston-Parmar spline model, by extending the CoxPHFitter python package to allow prediction using the odds scale or the hazard scale, analogous to the flexsurv R package.^22^ The optimum spline model, which was implemented for comparison of prediction performance with the optimum DL model, was using the odds scale with two nodes, analogous to.^11^

Hyperparameter tuning of the DL model used a composite objective combining median absolute error (MAE, 3 × weight) and AUROC (1 × weight) via a Weighted Normalised Sum (Supplementary Fig. S1). This prioritised MAE while retaining classification performance signal. Final model selection was based on a balanced compromise across MAE, AUROC at 12 months, and concordance, using the TOPSIS algorithm,^23^ with equal weighting. Hyperparameter tuning focused on concordance *only* does not significantly improve concordance or AUROC, but does degrade MAE performance (Supplementary Fig. S3). The predicTTE app provides capacity to select alternative outcome measures and/or weighting in future use-cases.

#### Training and testing optimal models

After model choice and hyperparameter tuning, the optimal model (determined by hyperparameter tuning) was trained using 10-fold cross validation similar to in model selection and hyperparameter tuning. In each fold 80% of training samples were selected at random for model training and 20% of training samples are used for evaluation. After 10 folds, the final prediction is output as the median of the 10 different predictions. The C-index (concordance) was calculated based on^24^ and in case of ties in predictions and event times adjusted according to.^12^ Model evaluation was ultimately carried out in the two external validation cohorts.

The baseline model for prediction of time from diagnosis to requirement for gastrostomy was trained using age of disease onset, presence/absence of an ALS-associated *C9orf72* mutation,^25^ weight at diagnosis and presymptomatic/premorbid weight, site of disease onset, diagnostic delay, ALSFRS-R slope, forced vital capacity (FVC, percentage of predicted based on normative values for age, sex, body height), cohort or geographical location of clinical care, and sex. For the longitudinal model which was retrained for patients who did not require a gastrostomy at 6 months after diagnosis, additional covariates included were the slope of the change in the weight and the timing of a weight measurement at approximately 6 months (range 4 and 8 months) after diagnosis.

#### Training and testing the MissForest model for imputation of missing data

A key aspect of our platform is the capability to impute missing data using a model called “MissForest”, which has shown superior performance in real-world testing^26^ and an ability to simultaneously handle continuous and categorical data.^27^ MissForest imputes data iteratively, starting with the variable with the least missing observations and progressing to the variable with the most missing observations. A random forest model is fit on the observed values. Each imputed value in this study relied on the mean result from 10 rounds of imputation because this represented the best compromise between computational time and independence of initial random seeds. For testing predictions within the external validation dataset, the imputation model was fit to the training dataset with pre-imputed data, plus an individual test patient from the validation dataset. In this way we avoid data leakage from the external validation dataset. As an extra test we randomly selected and omitted 150 data points from each covariate in the training dataset although we avoided removing more than two data points from any single patient; to avoid bias from incorrect information this analysis only included uncensored patients (Supplementary Fig. S4).

### Role of funders

Funders specified in the Acknowledgements section, had no role in the study design, data collection, data analyses, interpretation, or writing of reports.

## Results

### Choice of clinical measurements for prediction of time between diagnosis and gastrostomy requirement

To train a model for prediction of the timing of future gastrostomy requirement, as indicated by 5% weight loss from diagnosis,^6^ we chose a set of baseline clinical variables that have previously been associated with the rate of ALS progression^11^: age of disease onset, presence/absence of an ALS-associated *C9orf72* mutation,^25^ site of disease onset, diagnostic delay, ALSFRS-R slope, forced vital capacity (FVC, percentage of predicted based on normative values for age, sex, body height), and cohort or geographical location of clinical care. Diagnostic delay is the time from symptom onset to diagnosis with ALS and has been consistently linked to ALS survival,^28^ probably because it represents the speed of progression to the point where the disease is both clinically manifest and the patient has sufficient functional impairment to seek medical assistance. The ALSFRS-R is a commonly used functional rating scale for ALS^29^; to infer the rate of change or ‘slope’ we assumed a linear decline between the time of symptom onset and the time of diagnosis i.e. over the period which constitutes the diagnostic delay. Importantly all of these data are frequently collected at ALS diagnosis. We also added sex because there is evidence that sex impacts ALS biology,^30^ weight at diagnosis, and presymptomatic/premorbid weight. Site of onset explicitly includes bulbar onset including symptomatic dysphagia which is associated with requirement for gastrostomy. However, we removed patients who required gastrostomy at baseline i.e. where prediction was not necessary.

Cohorts used for model training and evaluation were broadly similar in the proportion requiring gastrostomy (Supplementary Fig. S1a), the rate of disease progression (Supplementary Fig. S1b), the proportion of missing data (Supplementary Fig. S1c) and the distribution of model covariates (Supplementary Fig. S1d). The exception was slightly more rapid progression in the Swedish validation cohort (Supplementary Fig. S1b) and the fact that presymptomatic/premorbid weight was missing from both of the external validation cohorts (Supplementary Fig. S1d). Heterogeneity in cohorts is a useful test of the generalisability of model performance. We note that there is a non-linear correlation between rate of change in weight for patients who required gastrostomy during the observed period, but no correlation at all for patients with censored data (Supplementary Fig. S1e and f). Therefore censored patients were not used for assessment of absolute time to gastrostomy requirement, although it is possible to use these patients to assess relative measures such as concordance.

### Optimised model choice and hyperparameter tuning

Next we sought to choose an optimal model for prediction of the timing of future gastrostomy requirement using our chosen baseline clinical measurements. We implemented >5000 different model configurations including spline models^11^ and a set of non-linear DL models designed for time-to-event prediction.^12^ To avoid overfitting we implemented a Bayesian framework for model tuning^16^ (**Methods**). We applied our optimised hyperparameter tuning to our training data including 3000 patient profiles from within the PRECISION^13^ dataset, taken from eight sites across Europe. For prediction of time from diagnosis to requirement of gastrostomy a logistic hazard DL model^19^ produced the optimal performance in 20% held-out test data (Supplementary Table S1, Supplementary Fig. S2b and c).

### Prediction performance is stable in internal cross-validation and external validation cohorts

**Fig. 2:**
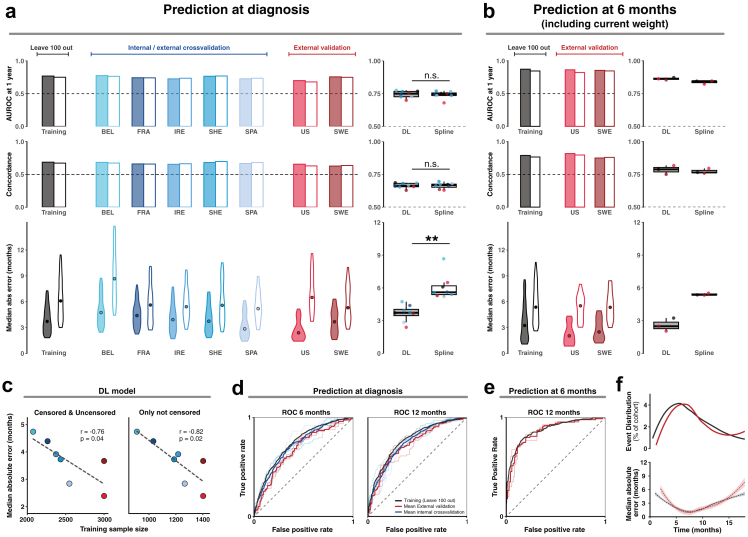
**Prediction of time to gastrostomy is stable in internal and external validation**. (**a**) Prediction performance for time from diagnosis to requirement for gastrostomy is evaluated in the PRECISION training dataset (n = 3000) via leave-100-out cross validation (left panel) and leave-one-cohort out cross validation (middle panel); and in two separate external validation cohorts (United States, n = 299; and Sweden, n = 215, right panel). Model performance is shown for AUROC at 12 months from diagnosis (upper panels), concordance (central panels), and the median absolute error between observed and predicted time from diagnosis to requirement for gastrostomy (MAE) (lower panels). p-value by rank sum test; ∗∗p < 0.01. (**b**) For patients who did not require a gastrostomy at six months after diagnosis a new longitudinal model is trained and evaluated including observed weight at ∼6 months. Violin plots are used to illustrate MAE (circle) together with the interquartile range. (**c**) Model performance by MAE is plotted against the number of training samples for both censored and uncensored observations. (**d-e**) ROC curves for the initial prediction of time from diagnosis to gastrostomy (**d**) and in the updated longitudinal model (**e**). (**f**) Recorded times to requirement for gastrostomy are binned by month and plotted to show the distribution through the cohort (upper panel) with the corresponding model performance by MAE (lower panel). Dark shading indicates the optimal logistic hazard deep learning (DL) model; light shading indicates the optimal spline model. BEL = Belgium, FRA = France, IRE = Ireland, SHE = UK, SPA = Spain, PRO-ACE = USA, SWE = Sweden. In all panels, colour indicates cohort.

Next we evaluated performance of the optimal logistic hazard DL model for the timing of future gastrostomy requirement, via internal cross validation and then in two external cohorts which were not utilised in hyperparameter tuning. We compared the performance of the optimal logistic hazard DL model to an optimal spline model because a Royston-Parmar spline model has previously been used successfully to predict ALS survival time.^11^ First we trained both models and performed internal cross validation using held-out cohorts from the PRECISION dataset (n = 3000) (Fig. 2a, left and middle panels); before then testing in two external validation cohorts from the US (PRO-ACE, n = 299) and Sweden (Karolinska, n = 215) (Fig. 2a, right panels). For the optimal logistic hazard DL model, AUROC for the presence/absence of gastrostomy requirement at 12 months after diagnosis was 0.75 (Fig. 2a, upper panels and Fig. 2d) and concordance was 0.67 (Fig. 2a, middle panels) for all cohorts; and performance of the optimal spline model was not significantly different to the DL model (Fig. 2a, upper and middle panels). For the optimal logistic hazard DL model, in internal cross validation the MAE was 3.7 months with some variability between cohorts (Fig. 2a, lower panels); here the optimal logistic hazard DL model was clearly superior to the optimal spline model (Fig. 2a, lower panels) and this difference was statistically significant (rank sum test, p = 8e-3). In the external validation the DL model achieved a MAE of 2.2 months in the US cohort, and 3.4 months in the Swedish cohort (Fig. 2a). Performance in the Swedish cohort is in-line with other cohorts despite a distinct clinical profile (Supplementary Fig. S1b). Performance metrics are provided in Supplementary Table S2.

We hypothesised that model performance might be improved by incorporating longitudinal data. We repeated model choice and hyperparameter tuning for a new model, using the same training data but with the addition of weight measurement at ∼6 months (range 4–8 months based upon available data, Supplementary Fig. S1e and f). Again the optimal model was a logistic hazard DL model (Supplementary Table S1). For patients who did not require gastrostomy before six months, this longitudinal DL model significantly improved prediction performance (MAE 2.6 months, AUROC at 12 months 0.86, concordance 0.79) (Fig. 2b–e, Supplementary Table S2); again there was a significant difference in prediction performance for MAE between the optimal logistic hazard DL model and an optimal spline model (rank sum test, p = 0.02). Comparison of performance of the optimal logistic hazard DL model with training and testing within individual cohorts demonstrates that MAE improves linearly as a function of the number of training samples (Fig. 2c) which supports efforts to add additional training data.

The distribution of time to gastrostomy is not uniform (Fig. 2f, upper panel). In the training cohort 32% of patients required a gastrostomy between 5 and 10 months after diagnosis and in the external validation cohorts this proportion was 48%. If we consider both the baseline and the longitudinal model predictions together, the optimal DL model achieves MAE of 1.2 months for these patients (Fig. 2f, lower panel, Supplementary Table S2).

Prediction performance was relatively stable across clinical subgroups. We divided patients by sex, site of onset and rate of change in the ALSFRS-R and examined the difference between predicted and actual time to requirement for gastrostomy (Supplementary Fig. S6). Prediction performance was degraded for patients with a slower (<1 point per month) rate of change in the ALSFRS-R, which reflects the sparsity of training data. However, the critical event we want to avoid is late identification of requirement for gastrostomy and the model performs particularly well with <6 months error for *all* patients with a faster (>1 point per month) rate of change in the ALSFRS-R.

We conclude that an optimally trained logistic hazard DL model is potentially able to achieve a level of performance which is accurate and stable enough to guide clinical decision making for *individual* patients with ALS.

### Data missingness is frequent and can be imputed using MissForest

Reported results above include imputation of missing covariates via a random-forest model called ‘MissForest’.^27^ Missing data is a common real-world phenomenon, which necessitates the adoption of imputation methods that can yield highly accurate results. Moreover the clinical progression profile of patients with ALS with missing data is not equivalent to those without missing data^31^ and thereby, a model that neglects patients with missing data does not capture the full range of ALS phenotypic variation. Numbers and proportions of missing data used in training are detailed in Supplementary Fig. S1.

**Fig. 3:**
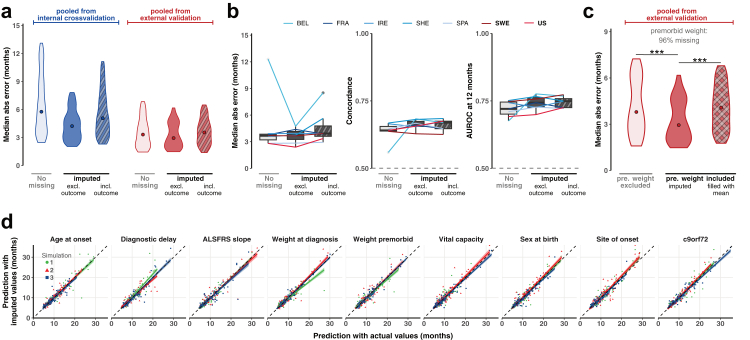
**Missing datapoints can be imputed using MissForest without degrading prediction performance**. (**a**) Median absolute error between observed and predicted time from diagnosis to requirement for gastrostomy (MAE), consisting of the pooled predictions taken from leave-one-cohort-out cross validation in the training dataset (left, blue), and the pooled predictions in the external validation dataset (right, red). Performance is shown after removing all patients containing missing data (light shading) or imputing missing data (dark shading), either using no outcome measurement (no pattern) or observed event times (diagonal stripes). (**b**) Performance evaluated by MAE, concordance and AUROC at 12 months is shown for all 7 cohorts instead of the pooled data using the same approach as in (**a**). (**c**) In the pooled external validation dataset 96% of the patients had no record of presymptomatic/premorbid weight. The MAE is shown comparing a model, where in training and validation premorbid weight was excluded (light shading), imputed, or filled with the mean presymptomatic/premorbid weight of the training cohort. ∗∗∗p < 0.001 rank sum test. (**d**) Prediction of time from diagnosis to gastrostomy requirement in the training cohort is compared using actual or imputed values of each covariate. 150 observations (10% of uncensored values in the training cohort) were randomly removed and imputed to generate predictions, and this procedure was repeated in three different simulation rounds. Predictions were performed using the optimal DL model. Predicted values are shown until 95th quantile, colours indicate the simulation round.

MissForest was trained using the relationships between observed covariates in the training dataset (PRECISION, n = 3000 of whom n = 626 had no missing data). In both internal cross validation in the training cohort (Fig. 3a, left panel), and in the external validation cohort (Fig. 3a, right panel), imputation of missing data improves model prediction performance as measured by MAE. Heterogeneity in missing data between cohorts prohibited evaluation using individual cohorts (Fig. 3b), and therefore we pooled cohorts in evaluation of model performance (Fig. 3a–c). We noted that presymptomatic/premorbid weight was missing almost entirely from both external validation cohorts (Supplementary Fig. S1). We wondered if a covariate could be entirely imputed and how this would impact model performance. Strikingly, in external validation, a model with imputed presymptomatic/premorbid weight significantly outperforms a model not trained with presymptomatic/premorbid weight, or one where missing presymptomatic/premorbid values have been replaced with the cohort mean (Fig. 3c). This suggests that the optimal DL model is able to learn relationships between covariates, and that imputation adds useful information for prediction performance.

An important decision in imputation of missing data is whether to include the outcome variable in imputation. Inclusion can increase the risk of overfitting to the training set whereas omission can artificially depress the importance of imputed data points.^32^ However, if the missingness of the covariate is a determinant of the relationship between the covariate and the outcome variable (e.g. if those with rapid disease progression are less likely to perform all tests) then imputing including the outcome variable relies on an incorrect assumption.^33^ To evaluate these alternatives we performed imputation, training and prediction under both scenarios: with and without the use of the outcome variable in the imputation of missing datapoints; here the outcome variable is the *uncensored* timing of gastrostomy requirement. In both internal cross validation in the training cohort (Fig. 3a, left panel) and in the external validation cohorts (Fig. 3a, right panel), imputation of missing data without the outcome variable improves model prediction performance.

To further test the performance of the MissForest model we randomly selected and omitted one covariate value from each of 150 patients (10% of non-censored patients) within the training dataset, in addition to existing missing data. These data were then imputed using the MissForest model and used to train the optimal DL model to predict time from diagnosis to requirement for gastrostomy. This procedure was repeated with three different random selections of covariates, but we avoided omitting two covariates from any single patient in each instance. For all covariates there was a significant correlation between predicted time to gastrostomy requirement with actual values and with imputed values (Fig. 3d). Moreover, imputed values are strongly correlated with the values they replaced (Supplementary Fig. S4) supporting the efficacy of our imputation strategy.

### The full set of clinical covariates contribute to prediction model performance

**Fig. 4:**
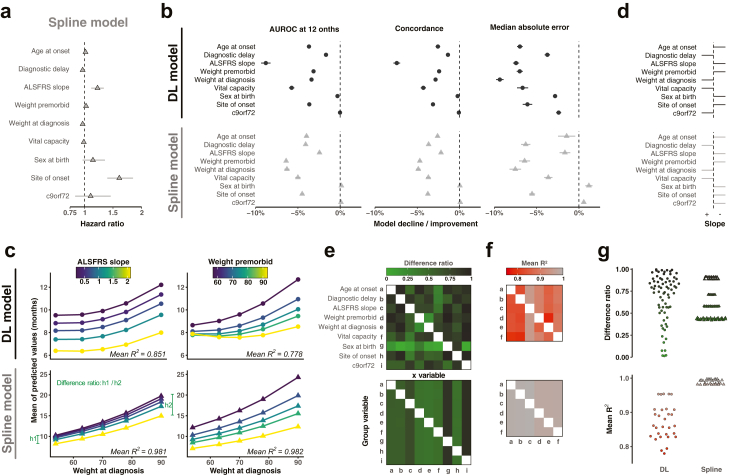
**Optimal DL model performance is determined by a non-linear combination of covariates**. (**a**) Hazard ratios (and 95% confidence interval) for each covariate are calculated from the optimal spline model used predict time to requirement for gastrostomy in the training data (n = 3000). (**b**) Prediction times produced using the optimal DL model (top panels) and the optimal spline model (lower panels), were evaluated for the training dataset and 50 simulated datasets where each of the ten covariates was permutated five times. Percentage of improvement or decline in model performance was calculated by comparing to non-permutated data; positive values indicate improved model performance. Shown is the mean (circle) ± SEM (error bar) for each of the five permutations per covariate. **(c**–**g)** A completely simulated dataset of 187,500 patients was created by combining every possible combination of observed values of the ten covariates; to achieve this numerical covariates were categorised into quintiles. The trained optimal DL and spline models were then used to make predictions for time to gastrostomy requirement using this dataset. (**c**) To consider the interaction between weight at diagnosis and ALSFRS-R slope (left panels) or presymptomatic/premorbid weight (right panels), mean predicted time to gastrostomy is plotted for all possible combinations of these covariates. R^2^ is calculated by fitting a linear model for each combination, and the difference ratio (h1/h2) captures the variability in model predictions as a function of weight at diagnosis. Similar linear models are fitted for all possible covariate pairs: (**d**) The slope of linear models demonstrates the direction of interdependence where a negative slope indicates that a higher value of the covariate is associated with early gastrostomy requirement. (**e**) Difference ratio and **(f**) mean R^2^ values for all possible pairs of covariates using the optimal DL model. (**g**) Distribution of the difference ratios (top) and the mean R^2^-values (bottom) for both the optimal DL and optimal spline models.

We wanted to determine which covariates are important for prediction model performance. A theoretical advantage of a DL model is that it is capable of extracting knowledge from non-linear combinations of covariates. This contrasts with an additive linear model where each covariate contributes a fixed value to prediction performance. For example, applying a linear Cox proportional hazards model to prediction of time from diagnosis to requirement for gastrostomy demonstrates that performance is dictated by site of disease onset, ALSFRS-R slope and sex (Fig. 4a). To investigate the relationship between covariates and model performance for the optimal DL prediction model we created a simulated dataset derived from our original training data. Each of the ten covariates was permutated randomly in a replica of the original 3000 patients, and this was repeated five times for each covariate (total of 150,000 simulated patients). We could then determine the effect on performance of the trained model of permutating each covariate: For all outcome measures and in both the optimum spline model and the optimal logistic hazard DL model, almost all covariates impact model performance (Fig. 4b). For MAE and the optimal logistic hazard DL model then *all* covariates are important (Fig. 4b, right upper panel).

Next, we examined the relationship between predicted time from diagnosis to gastrostomy requirement as it depends upon *combinations* of any two other covariates. The idea was to look for evidence of non-linear interactions between covariates. To do this we simulated a new dataset including 187,500 instances; simulated patients were given all possible combinations of quintiles for each of the six numerical covariates, and both possible values for binary covariates. We used our trained models to generate a predicted time to gastrostomy requirement for each simulated patient and examined how systematically altering each covariate impacted the *value* of the model prediction. We find evidence that both the optimal spline model and the optimal logistic hazard DL model capture interactions between covariates (Fig. 4c). Notably, *C9orf72* genetic status has an opposite effect on prediction of time to event in the DL model compared to the spline model (Fig. 4d). Using the interaction between ALSFRS-R slope or presymptomatic/premorbid weight and weight at diagnosis as an example, for the DL model there is evidence that covariate interactions are non-linear (Fig. 4c, upper panels) but for the spline model the interactions are linear (Fig. 4c, lower panels). The same is true for all combinations of covariates (Fig. 4e–g, Supplementary Fig. S5).

### Webpage implementation of predicTTE

predicTTE is a self-contained software package designed to facilitate the design and training of time-to-event prediction models. We have provided access to our trained model for prediction of time to gastrostomy requirement through an accompanying online portal (www.predictte.org) designed to be used by researchers and clinicians without any requirement for computational expertise. Users of the portal, including clinicians, can also contribute their own data for model training (Fig. 1) and access our app to design and train their own models. Data submitted within the portal is anonymised and securely stored.

## Discussion

Accurate prediction to enable timely gastrostomy for patients with ALS has the potential to significantly improve clinical care, optimise healthcare resource planning and improve eligibility criteria in clinical trials of oral drugs. We provide a new optimal deep learning (DL) model for prediction of time from ALS diagnosis until requirement of gastrostomy. Model covariates are measurements routinely collected at diagnosis including age, sex, site of disease onset, the length of time required for diagnosis and geographical location; plus weight measured at 6 months. We employed a training dataset of 3000 patients from Europe, and two external validation cohorts spanning distinct populations and clinical contexts (United States, n = 299; and Sweden, n = 215). Missing data was imputed using a random forest model. Our model achieves a new benchmark for accuracy, with stable performance in external validation cohorts sourced from distinct populations and clinical contexts; the median error between actual and predicted time to requirement for gastrostomy is just 1.2 months in the largest group of patients. Using synthetic data we conclude that model performance is contributed by all covariates and non-linear combinations of covariates.

Our optimal prediction model provides individualised predictions of the timing of future gastrostomy requirement which are potentially accurate enough to guide clinical decision making. In the majority, training and validation cohorts reflect ‘real-world’ clinical data, and prediction performance is stable across multiple cohorts, populations and clinical contexts suggesting that it is generalisable. Absolute prediction time could be used to provide an individualised target for gastrostomy preparation; or the binary prediction as to whether gastrostomy will be required at 12 months after diagnosis could be used to prioritise immediate versus delayed gastrostomy planning.

We chose to use 5% weight loss from diagnosis as an objective measure of the time when gastrostomy should be considered, although we accept that the decision to actually perform a gastrostomy will include additional considerations, notably patient preference. Here we are following the recommendations of a prospective cohort study.^6^ This study noted that more than 10% weight loss from diagnosis at the time of gastrostomy was associated with reduced survival, and that refractory cachexia is likely to occur after 5% weight loss from diagnosis, based upon research in oncology.^34^ The idea of our work is to provide prediction to facilitate early intervention and therefore we focused on the 5% cut off. This will also increase the probability that the 10% threshold is *not* reached within the error inherent in our prediction times. Weight loss in patients with ALS can occur without significant bulbar weakness, for example due to disease-associated hypermetabolism,^35^^,^^36^ reduced appetite or impaired physical capacity to access nutrition. In this scenario extra calorie intake could be provided orally but, in the context of an ALS diagnosis, significant disability is likely to be present and as a result there is still a clinical indication for gastrostomy. We accept that the weight loss criteria we propose will not encompass every possible scenario and patient-specific decision making is essential.

We provide an implementation of a random forest model—MissForest—for imputation of missing data. In testing this method, we considered whether the outcome variable should be used in imputation. We show that inclusion of the outcome variable can degrade model performance in a validation dataset. This is in opposition to previous findings^37^ but notably the contradictory study predates the development of the set of spline and DL time-to-event prediction tools we have applied. In particular, DL models may be more vulnerable to overfitting when missing values within the training data carry an artificial signature of the outcome variable. Our imputation of missing data is sufficiently effective that imputation of presymptomatic/premorbid weight improves model performance, even when this variable is not recorded in the external validation cohort. We also provide a new approach for hyperparameter tuning and model selection based on a weighted combination of outcome variables and Bayesian optimisation.

Our optimal DL prediction model is superior to an optimal spline model in discrimination but particularly in calibration, as demonstrated by the accuracy of absolute prediction values measured by the MAE. We provide evidence that this is because of non-linear dependencies captured by the DL model which are not captured by an optimal spline model. In the context of ALS, provision of an exact prediction time value aligns with expressed patient preference.^11^ A practical advantage of an exact prediction value is that results are optimally portable to other applications such as a study of genetic drivers of ALS rate of progression.

Our predicTTE framework, including our app and online portal, are designed to widen accessibility to optimal time to event prediction models. The aim is to provide cutting-edge computational tools to non-expert users who hold the appropriate clinical data. Rapid development in the statistical tools for time-to-event prediction has had limited impact because of the requirement for advanced statistical knowledge and computational expertise. Improvements in prediction will likely provide accurate prognoses, guide personalised medicine and facilitate timely clinical interventions. Moreover, our secure online portal enables sharing of data and distribution of individualised prediction to researchers and clinicians, who can then contribute their own data via the online portal. We envision a positive feedback loop leading to exponential improvements in training data size and prediction model performance.

### Caveats and limitations

A limitation of our work is that we were not able to test our predictions in cohorts from outside of Europe and the US. Conclusive demonstration of clinical utility, which is related but distinct from accurate prediction of gastrostomy requirement, will require a prospective study. We have shown that our predictions are valid across the clinical spectrum of ALS (Supplementary Fig. S6) but we acknowledge that certain subgroups, such as ‘flail limb’ presentations, are under-represented in our data and we cannot yet be certain that they will be well served by our model.

## Contributors

MW, OH, CMD and JCK conceived and designed the study. MW developed predicTTE. MW, HMD, AE, SB and JCK performed statistical analyses. PRECISION ALS is led by AC, AM, AH, CI, JVC, LvdB, MP, PvD, PC, MdC, AAC, PJS and CMD. Additional data was collected by NY, IM, YC, CH, SG, JA, JMK, KPK, SZ, AI, AAK, PMP, and EH. MH and EMD curated PRECISION ALS data. MW, HMD, CH, AE, SB, CMD and JCK interpreted the data with assistance from all other authors. OH, CMD, PJS and JCK supervised the work. MW and JCK wrote the manuscript with feedback from all other authors. All authors read and approved the final version of the manuscript. MW and JCK accessed and verified the underlying data.

## Data sharing statement

Data used in this study is available to researchers via application to PRECISION ALS (https://www.precisionals.ie/contact-us/),^13^ or PRO-ACE (https://www.data4cures.org/requestdata).^14^ The predicTTE online portal (https://www.predictte.org/) includes instructional material and links to download the app for different platforms.

## Declaration of interests

Adriano Chiò serves on the editorial advisory board of Amyotrophic Lateral Sclerosis and Neurological Sciences. Adriano Chiò serves on scientific advisory boards for Mitsubishi Tanabe, Biogen, Roche, Denali Pharma, Cytokinetics, Lilly, Ferrer, Zambon Biotech, and Amylyx Pharmaceuticals, has received a research grant from Biogen and serve on Drug Safety Monitoring Board for AB Science, Corcept, and Eli Lilly. He has received research support from the Italian Ministry of Health (Ricerca Finalizzata), Regione Piemonte (Ricerca Finalizzata), Italian Ministry of University and Research (PRIN projects), University of Turin, and the European Commission (Health Seventh Framework Programme, Horizon 2020 and Horizon Europe).

Andreas Hermann has received personal fees and non-financial support from ITF Pharma, Amylyx, Biogen, NRG therapeutics and Desitin. He has received royalties from Elsevier Press and Kohlhammer.

Caroline Ingre, Neuromuscular Disease Specialist, Head of the ALS Center at Karolinska University Hospital, ALS Research Group Lead at Karolinska Institute, Founder of the National ALS registry in Sweden and the Karolinska Treatment Center, Hosts of ENCALS ALS Meeting 2024, Sweden.

Leonard van den Berg, Professor of Neurology Director, ALS Centre, University Medical Centre, Utrecht, Chair, TRICALS Consortium, The Netherlands.

Mònica Povedano Panadés reports consultancies/advisory boards for Amylyx Pharmaceuticals, Biogen, Ferrer, Grifols, Italfarmaco, Mitsubishi Tanabe Pharma and Roche.

Philip Van Damme reports advisory boards for Biogen, CSL Behring, Alexion Pharmaceuticals, Ferrer, QurAlis, Cytokinetics, Argenx, UCB, Muna Therapeutics, Alector, Augustine Therapeutics, VectorY, Zambon, Amylyx (paid to institution). He has received speaker fees from Biogen, Zambon and Amylyx (paid to institution). He is supported by the E. von Behring Chair for Neuromuscular and Neurodegenerative Disorders (from CSL Behring, paid to institution).

Philippe Corcia reports consultancies or advisory boards for Amylyx, Biogen, Coave Therapeutics, Cytokinetics, Ferrer, Mitsubishi Tanabe, QurAlis, Vectory, Zambon. He is member of the Board of the Journal Amyotrophic Lateral Sclerosis and the Frontotemporal Dementias and of the Revue Neurologique.

Ammar Al-Chalabi reports consultancies or advisory boards for Amylyx, Apellis, Biogen, Brainstorm, Clene Therapeutics, Cytokinetics, GenieUs, GSK, Lilly, Mitsubishi Tanabe Pharma, Novartis, OrionPharma, Quralis, Sano, Sanofi, and Wave Pharmaceuticals.

Orla Hardiman reports consultancies/advisory boards for Biogen, Takeda, Ferrer, Novartis, Alchemab and Medici Nova. She is Editor in Chief of the Journal Amyotrophic Lateral Sclerosis and the Frontotemporal Dementias.

Juan Vazquez Costa is a member of the ERN-NMD and reports consultancies or advisory boards for Biogen and Roche.
