# Supplementary material for: Optimised machine learning for time-to-event prediction in healthcare applied to timing of gastrostomy in ALS: a multi-centre, retrospective model development and validation study
Source: eBioMedicine. 2025 Oct 10;121:105962. doi: 10.1016/j.ebiom.2025.105962 (PMC12547709; doi:10.1016/j.ebiom.2025.105962)

Supplementary Material Legends

Supplementary Figure 1: Training and external validation cohorts used for training and testing model prediction. **(a)** Overview of patient numbers across all cohorts. The training cohort (black) comprises seven sub-cohorts, with the upper five used for internal cross-validation (blue). Two external validation cohorts are shown in red. Dark shading indicates the number of uncensored patients where time to gastrostomy requirement was *not* observed, while light shading represents censored cases. Overall, approximately half of all patients were censored. **(b)** Kaplan–Meier curves for each cohort, color-coded as in (**a**). The right panel focuses on the critical time window during the first two years post-diagnosis. The two external validation cohorts span both extremes of survival, suggesting good generalisability. **(c)** Overview of missing data across all covariates and cohorts. Dark shading indicates missing values. Vital capacity and presymptomatic/premorbid weight show notably higher rates of missingness, with premorbid weight missing in ~96% of patients in both external validation cohorts. **(d)** Distributions of covariates across cohorts. Dashed lines represent the full data range, error bars indicate the interquartile range, black circles mark the median, and red diamonds denote the mean. "Site of onset" was treated as a categorical variable, while "sex at birth" and *C9orf72* mutation status were considered binary. Category proportions are color-coded as shown in the legend. **(e, f)** Left panels: Correlation between time to gastrostomy (x-axis) and weight slope—defined as the difference in weight from diagnosis to the closest available measurement at 6 months—for the training cohort (top, blue) and the external validation cohort (bottom, red). Uncensored patients are shown in blue/red, and censored patients in gray. Only patients who underwent gastrostomy or who passed the 6-month mark were included in the updated model (refer to **Fig. 2b**). Right panels: Timing of the closest weight assessment to the 6-month mark, color-coded by the weight slope. On average, weight measurements were taken around the 6-month point, with 50% of patients assessed between 5 and 7 months and 95% of all patients assessed between 4 and 8 months. There was no clustering of slope values based on assessment timing, supporting the validity of using the nearest measurement to 6 months in the longitudinal model.

Supplementary Figure 2: Hyperparameter tuning and model choice (**a**) Model selection flowchart. The tuning consists of three steps. First grid tuning where only 3 variables are varied but they are varied exhaustively, namely layer structure, the scheme and the intervals. The best three combinations of these variables are selected for the second step where 6 variables are optimised using a Bayesian approach: Optuna[16]. Here 100 combinations are tested using an Adam optimiser. In the third step, 3 more variables are optimised (9 total) and an AdamWR optimiser is employed to test 500 combinations. Optuna aims to minimise a combined feature consisting of the MAE and the AUROC at 12 months where the MAE is 3x upweighted compared to the AUROC. All three steps are repeated for each model type and finally, the best model configuration is selected for training and prediction.

(**b**) Comparison of performance in different outcome measures for all DL model types demonstrating that the optimal logistic hazard DL model performs best in a combination of all three measures. Density plots shown top and right. (**c**) Model performance over the three steps of hyperparameter tuning. Left panel: Choice of covariate combinations selectively improves performance over time (left to right) demonstrating that Optuna iteratively improves model performance without exhaustively testing all possible combinations of variables. Colour indicates DL model type as in (**b**). Right panel: Boxplot showing the same data grouped by the three steps of tuning, indicating the positive tendency and improvement from one step to the other, mainly for the chosen logistic hazard DL model and in particular with respect to MAE.

Supplementary Figure 3: Impact of outcome-based model selection on predictive performance across validation cohorts. Using a fixed set of model configurations (see **Methods; Supplementary Figure 2**), the final model was selected based either on a compromise across multiple outcome metrics—median absolute error (MAE), concordance index (C-index), and AUROC at 12 months—via the TOPSIS algorithm (dark shading), or based solely on maximising the concordance (light shading). Both resulting model configurations were evaluated using leave-one-cohort-out and external validation. Line colours represent individual cohorts, consistent with **Fig. 2**. While selecting the model based on concordance marginally improved both concordance and AUROC at 12 months, it led to a significant increase in MAE. These findings underscore that the choice of outcome metric used for model selection meaningfully influences final predictive performance, and that model selection strategies should be guided by specific clinical or research priorities.

Supplementary Figure 4: Effect of pairwise covariate interactions on predicted time to gastrostomy requirement. Each panel displays the mean predicted time to gastrostomy requirement (y-axis) from a simulated cohort of 187,500 patients, stratified across pairwise combinations of six numerical and three categorical covariates. For each panel, the x-axis represents one covariate, and the color indicates another, with points connected by lines to demonstrate the degree of linearity. Numerical covariates were represented using their empirical deciles, and categorical covariates by all observed levels. Colors for numerical covariates follow a continuous Viridis scale, while categorical variables use a fixed color scheme. Predicted times to gastrostomy requirement were generated using either the optimal DL model (first page) or the optimal spline model (second page).

Supplementary Figure 5: Agreement between original and imputed numerical values. Scatter plots illustrate the relationship between original (x-axis) and imputed (y-axis) values for all six numerical covariates, across three independent simulation rounds as in **Fig. 3**. Only observations with artificially introduced missing values (in test sets) and known ground truth (from the original dataset) are shown. Each point corresponds to a single observation, colored by test repetition. Solid lines represent least-squares regression fits; dashed lines denote the identity line ($y = x$) for reference.

Across all features a significantly positive correlation was observed in each repetition (overall mean correlation coefficient r = 0.543, ranging from r = 0.33 (age) to 0.79 (presymptomatic/premorbid weight).

Supplementary Figure 6: Prediction performance in clinical subgroups. Patients were divided into clinical subgroups based upon, sex, site of onset (limb or bulbar) and rate of change in the ALSFRS-R. ‘Fast’ designates patients with >1 ALSFRS-R point per month change over their observed disease course. ‘Slow’ designates patients with <1 ALSFRS-R point per month change over their observed disease course. The difference in actual and predicted time to gastrostomy requirement is shown for patients in the training cohort (**a**) and the external validation cohorts (**b**). Grey lines connect observed and predicted times for individual patients.

Supplementary Table 1: Hyperparameters for optimal prediction models

Supplementary Table 2: Performance metrics for all trained prediction models. For time dependent variables i.e. AUROC, Brier score and negative binomial, the ‘mean’ is determined by calculating the metric at month between 3 months and 3 years post-diagnosis taking the mean value; the idea is to show that the performance is stable over time. The ‘merged’ model included all patients but the predicted time to gastrostomy requirement was taken from the baseline model if gastrostomy occurred within the first 6 months, or the longitudinal model including weight measured at 6 months, if gastrostomy did not occur within the first 6 months.

Supplementary Figure 1

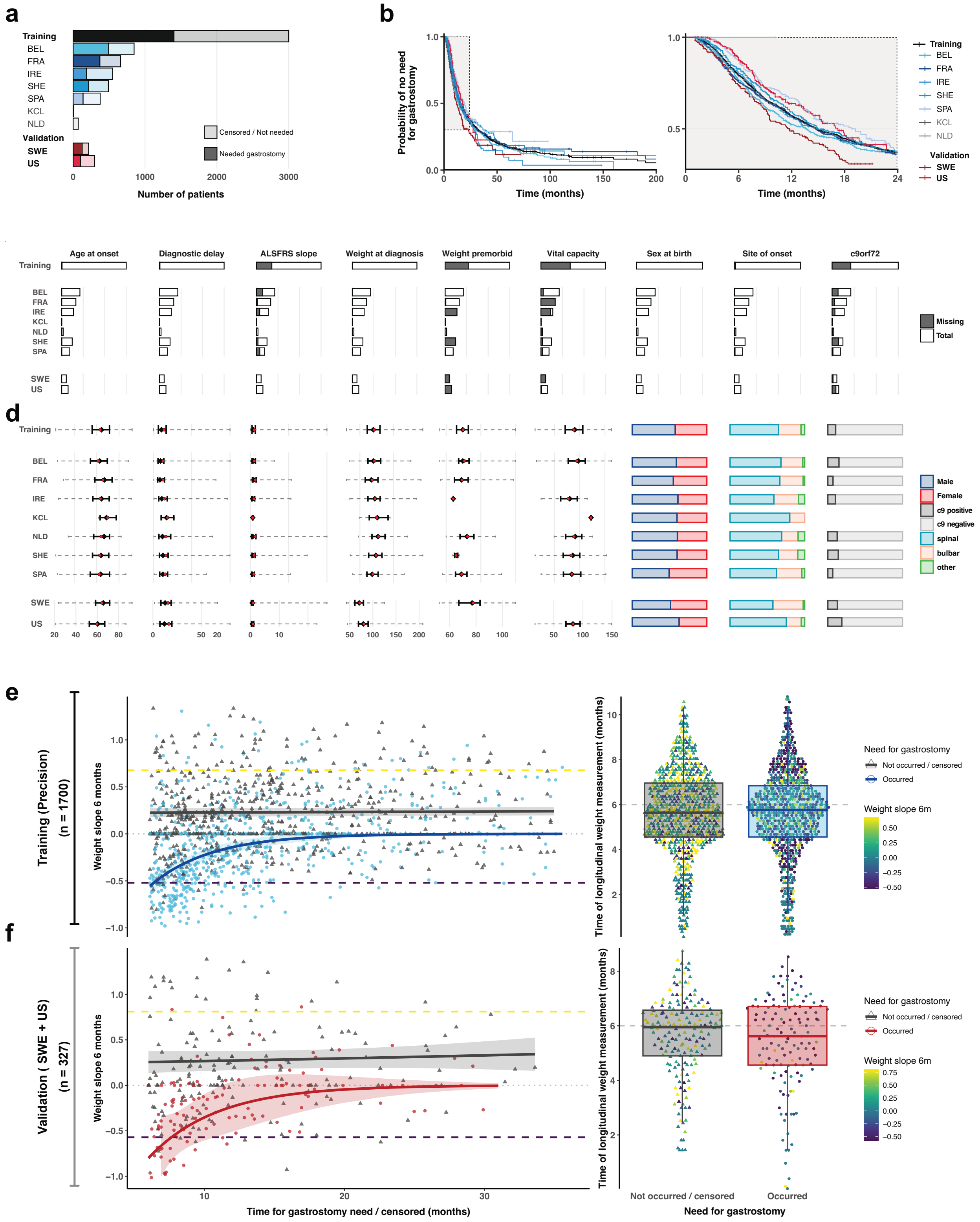

Supplementary Figure 2

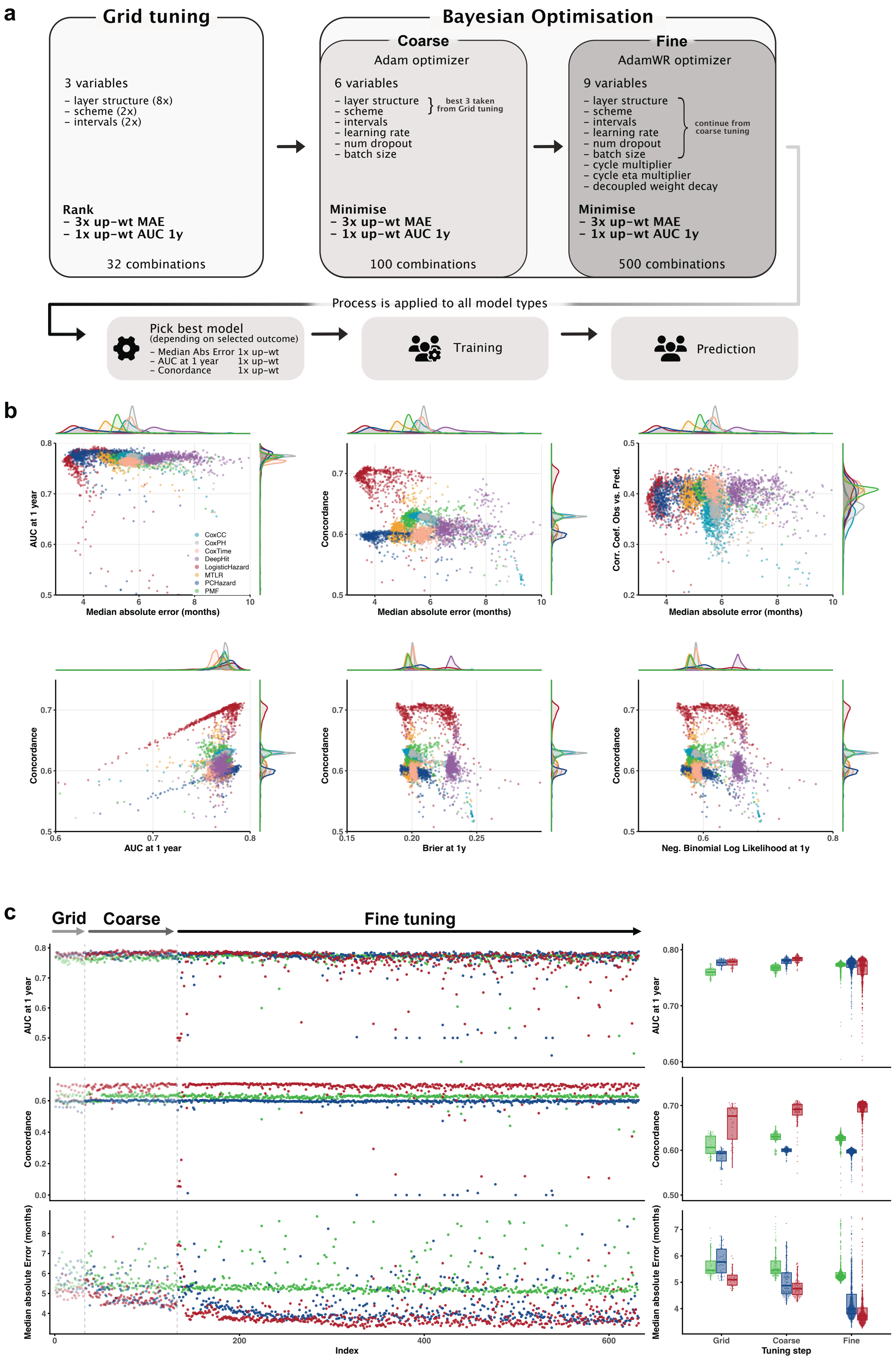

Supplementary Figure 3

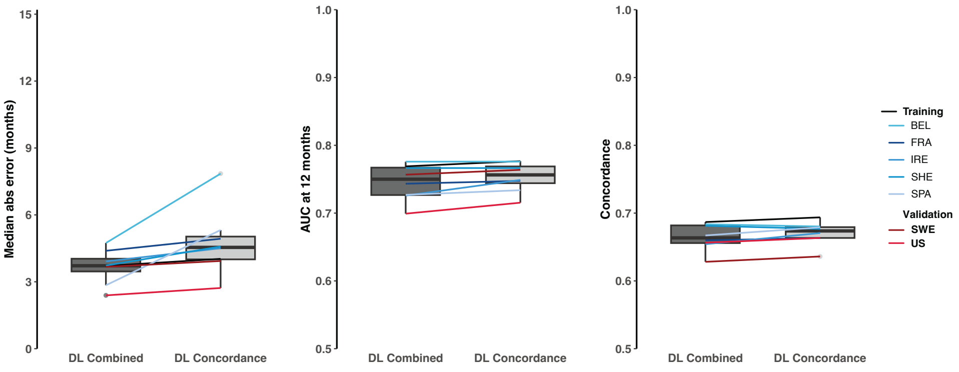

Supplementary Figure 4

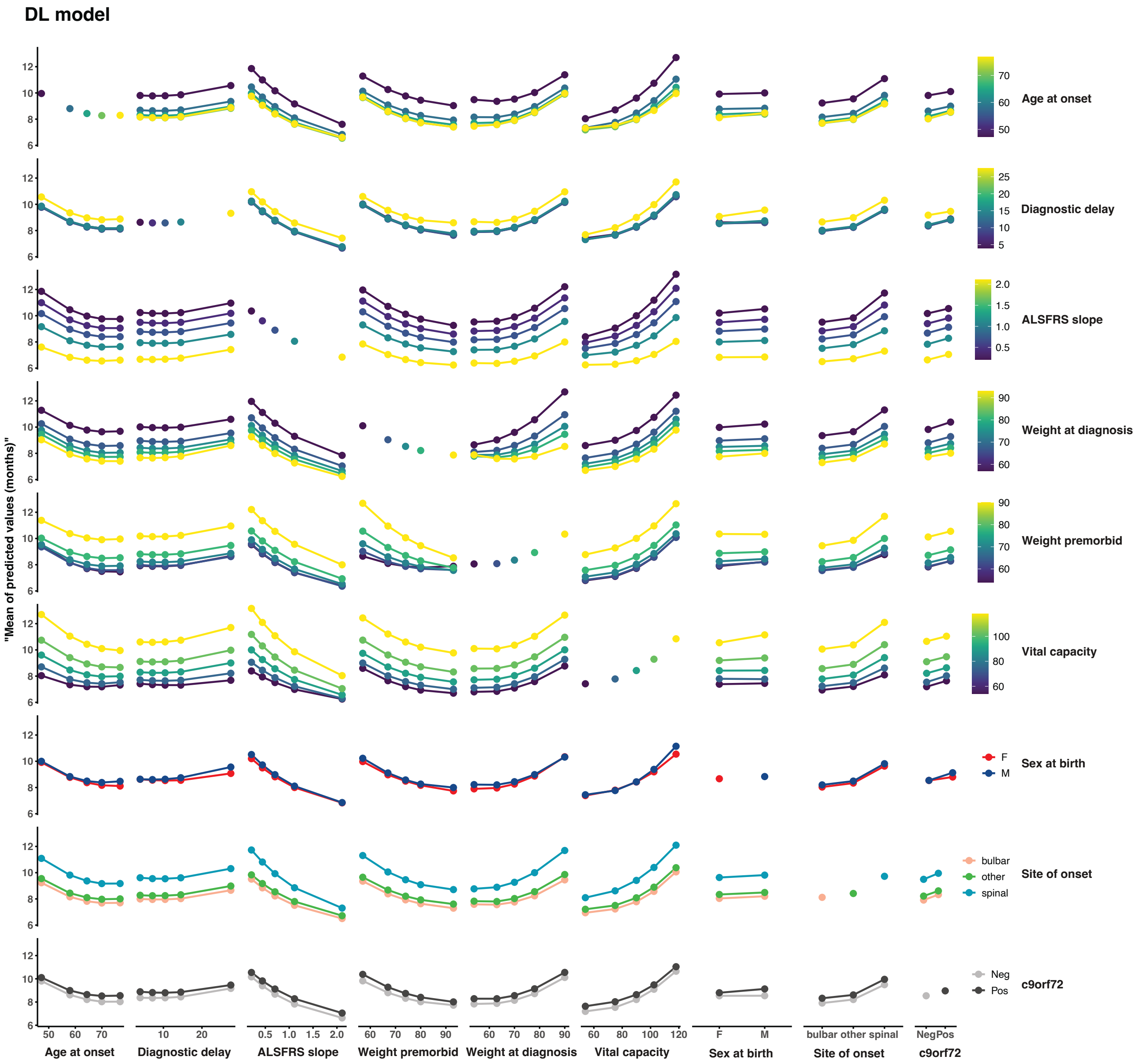

Supplementary Figure 4 (continued)

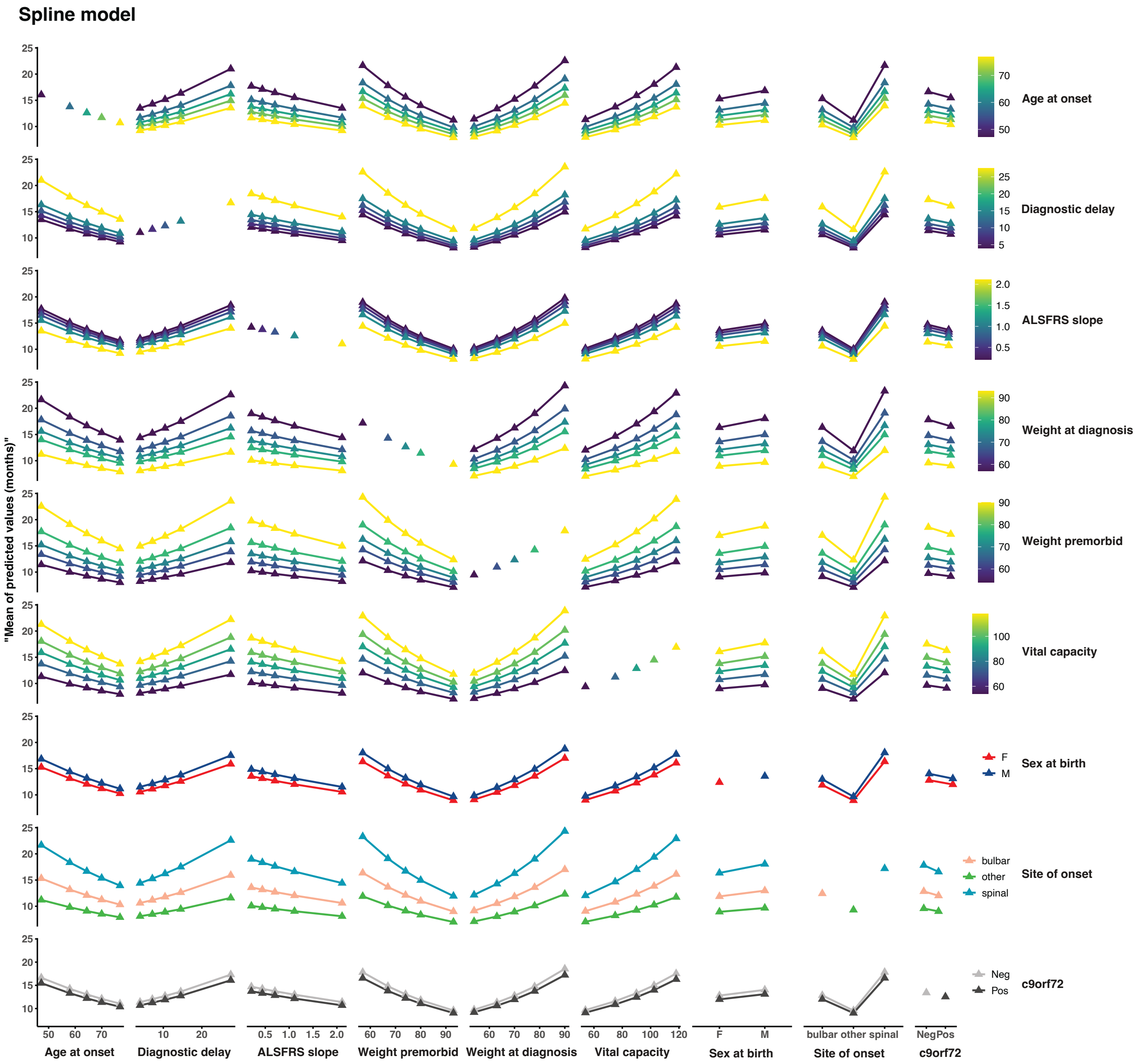

Supplementary Figure 5

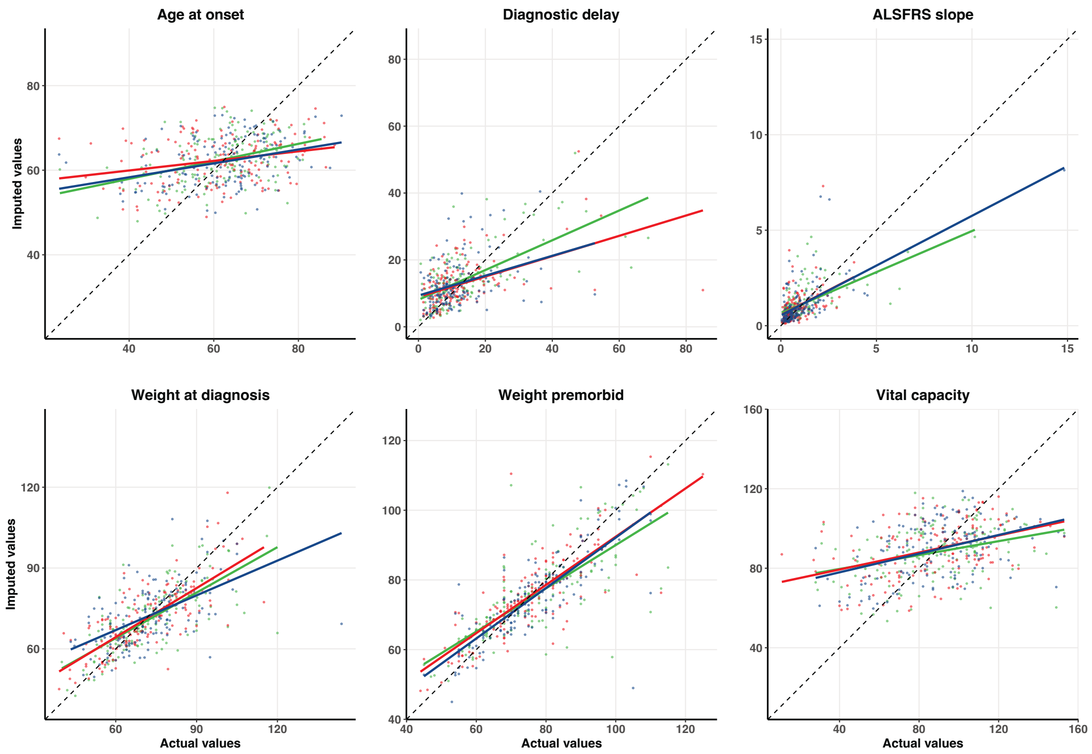

Supplementary Figure 6

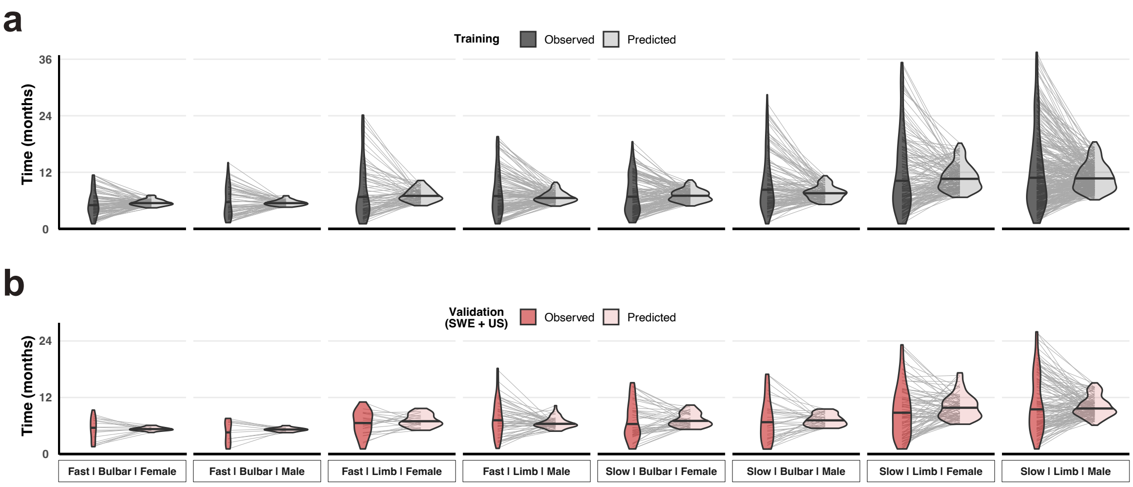

Supplement: Supplementary Figures [file mmc3.pdf]
